# Interaction between Chronic Inflammation and Oral HPV Infection in the Etiology of Head and Neck Cancers

**DOI:** 10.1155/2012/575242

**Published:** 2012-02-23

**Authors:** Mine Tezal

**Affiliations:** NYS Center of Excellence in Bioinformatics and Life Sciences, Department of Oral Diagnostic Sciences, State University of New York, Buffalo, NY 14214, USA

## Abstract

Incidences of oral tongue, base of the tongue, and tonsil cancers have been increasing steadily in many parts of the world in spite of declining rates of tobacco use over the last four decades. A better understanding of the etiology, interactions between risk factors, and new approaches to prevention and treatment are necessary to change this course. This paper will present evidence supporting a potential role of chronic inflammation in the etiologies of oral human papillomavirus infection and head and neck squamous cell carcinoma, and it will discuss the implications for prevention and treatment.

## 1. Introduction

Over 500 000 new cases of head and neck squamous cell carcinoma (HNSCC) occur annually in the world [1]. The National Cancer Institute's Surveillance, Epidemiology, and End Results (SEER) has reported a steady increase in the incidence of oropharyngeal cancers since 1973 despite the significant decline in tobacco use since 1965 [2]. Similar trends are observed in many parts of the world [3–5].

HNSCC is largely preventable, yet the majority of current research has focused on treatment and diagnosis. Most of the existing prevention strategies for HNSCC have been secondary and tertiary with limited success [6, 7]. Chemoprevention, including retinoids, selenium, vitamin E, interferon-*α* (IFN-*α*), cyclo-oxygenase-2 (COX-2), and epidermal growth factor receptor (EGFR) tyrosine kinase inhibitors, usually have obstacles including toxicity and reversal of their effects after cessation of treatment [8, 9]. Primary prevention strategies, focused mainly on smoking cessation, have not lead to a visible reduction in HNSCC incidence [1, 2]. Oral human papillomavirus (HPV) infection has emerged as an independent etiological factor for at least a subset of HNSCC, and is another target for primary prevention [10–12]. A highly effective vaccine is available to prevent cervical HPV infection which is currently recommended for females aged 9 to 26 years and males aged 9 to 21 years [13]. However, oral HPV infection can be transmitted at or any time after birth, and the target population for the vaccine to prevent oral HPV infection has not been defined [14]. A large percentage of the general population who are already exposed to the virus or those who already developed an HPV-related disease do not benefit from the current vaccine. A better understanding of the etiology of HNSCC, especially the interactions among risk factors, is critical for effective prevention strategies.

This short review will (1) present evidence supporting a potential role of chronic inflammation on the etiologies of oral HPV infection and HNSCC and (2) discuss the implications for prevention and treatment.

## 2. Chronic Inflammation and Head and Neck Squamous Cell Carcinoma

Clinical, epidemiologic, and animal studies support an association between chronic inflammation and cancer in several organs [15–18]. Chronic cervicitis and cervical cancer, ulcerative colitis and colorectal cancer, pancreatitis and pancreatic cancer, skin inflammation and skin cancer, esophagitis and esophageal cancer, and hepatitis and liver cancer are a few examples (Table 1).

In the oral cavity, periodontitis is a chronic inflammatory disease of the structures around teeth associated with dental biofilm [19]. The ensuing chronic inflammation leads to local pathologic anatomic changes, namely, periodontal pocket formation, clinical attachment loss (CAL), and alveolar bone loss (ABL) [20]. As the disease progresses and more bone is lost, junctional epithelium at the bottom of the periodontal pocket migrates apically along the root surface and the pocket depth increases. Untreated periodontitis eventually leads to tooth loss. The pocket epithelium is characterized by continuous proliferation, formation of rete-ridges, and ulcerations. In the connective tissue, there is increased angiogenesis, chronic inflammatory infiltrate, fibrosis, and connective tissue loss. The average prevalence of periodontitis in the general population is 30% [21]. Periodontitis results in a continuous release of inflammatory cytokines, enzymes, and toxins into saliva. The levels of these inflammatory markers in saliva is directly associated with the extent and severity of periodontitis [22, 23]. In addition, periodontal pathogens and inflammatory cytokines can travel with saliva and blood causing inflammation and tissue injury at distant sites [24–31]. Periodontal treatment has been shown to reverse some of these systemic adverse effects [32–34].

A link between poor oral hygiene and HNSCC has been suggested for over two decades [35–46] but the underlying mechanism of this association was not clear. Studies from our group have suggested that chronic periodontitis, an outcome of poor oral hygiene, is associated with oral premalignant lesions [47] and HNSCC [48, 49]. In a secondary analysis of the Third National Health and Nutrition Examination Survey (NHANES III), a large nationwide cross-sectional study, including 13,798 men and women of age 20 and older, a history of periodontitis, measured by clinical attachment loss (CAL), was significantly associated with the prevalence of oral premalignant lesions (OR = 1.55, 95% CI = 1.06–2.27) after adjusting for age, gender, race/ethnicity, education, tobacco, alcohol, and occupational hazard [47]. Periodontitis was not associated with the presence of any oral soft tissue lesion (OR = 1.09, 95% CI = 0.91–1.31) (Table 2) [47].

In a recent hospital-based case-control study with histologically confirmed 266 primary incident HNSCC cases and 207 controls in Upstate New York, ABL was significantly higher in cases compared to controls (4.04 mm versus 2.44 mm, *P* < 0.001). After adjustment for age, gender, race/ethnicity, marital status, smoking status, alcohol use, and missing teeth, each millimeter of ABL was associated with >4-fold increased odds of HNSCC (OR = 4.36, 95% CI = 3.16–6.01). The strength of the association was greatest in the oral cavity (OR = 4.52, 95% CI = 3.03–6.75), followed by oropharynx (OR = 3.64, 95% CI = 2.54–5.22) and larynx (OR = 2.72, 95% CI = 1.78–4.16) (Table 3). Furthermore, patients with periodontitis were more likely than those without periodontitis to have poorly differentiated oral cavity SCC (32.8% versus 11.5%, *P* = 0.038) [49].

The potential association between chronic inflammation and HNSCC is further supported by two recent case control studies suggesting a beneficial effect of nonsteroidal anti-inflammatory drugs (NSAID) against HNSCC [50, 51]. In a hospital-based case control study among 529 patients with HNSCC and 529 control subjects matched by age, sex, and smoking status, Aspirin use was associated with a 25% reduction in the risk of HNSCC (OR = 0.75; 95% CI = 0.58–0.96). Risk reduction was observed across all primary tumor sites, with cancers of the oral cavity and oropharynx exhibiting greater risk reduction [50]. In another more recent hospital-based case-control study among 71 incident HNSCC cases and 71 healthy controls, daily NSAID use was associated with 86% reduction in HNSCC risk (OR = 0.14; 95% CI = 0.04–0.54) after adjusting for educational level and marital status [51].

The biological mechanism of the association between chronic inflammation and cancer has been described extensively but is also evolving continuously since both inflammation and cancer are complex processes under the control of many driving forces [15–18]. Bacteria and their products, including endotoxins, enzymes, and metabolic by-products, may directly induce genetic and epigenetic changes in surrounding epithelial cells [52–54]. They also increase the production of carcinogenic acetaldehydes [55–57] and nitrosamines [58, 59]. However, the bulk of the available evidence supports an indirect association through stimulation of inflammation. Host cells, including neutrophils, macrophages, monocytes, lymphocytes, fibroblasts, and epithelial cells, respond to bacteria by generating (1) cytokines, chemokines, prostaglandins, growth factors, and other signals that provide an environment for cell survival, proliferation, migration, angiogenesis, and inhibition of apoptosis [60]. This environment helps epithelial cells to accumulate mutations, and drives these mutant epithelial cells to proliferate and migrate and gives them a growth advantage; (2) reactive oxygen species (hydrogen peroxide and oxy radicals), reactive nitrogen species (nitric oxides), reactive lipids and metabolites (malondialdehyde, 4-hydroxy-2-nonenal), and matrix metalloproteinases (MMPs) which can act as endogenous mutagens. Numerous *in vivo and in vitro* studies have confirmed the associations of several genes and proteins involved in different stages of inflammation with carcinogenesis [61–71]. In addition to its independent association with HNSCC, chronic inflammation may also act synergistically with other carcinogens to increase the risk of HNSCC. For example, breaks in the mucosal barrier due to chronic inflammation may lead to enhanced penetration of other carcinogens such as tobacco, alcohol, and dietary metabolites [72, 73].

## 3. Chronic Inflammation and Oral HPV Infection

The steady increase in the incidence of oropharyngeal cancers over the last four decades has been mainly attributed to oral HPV infection which has been accepted as an etiological factor for a subset of HNSCC [10–14]. HPV is a commonly transmitted virus but the majority of the infections are cleared rapidly by the immune system. Rather than its mere presence at one time point, persistence of the virus seems to be the critical factor for the development of HPV-related diseases [74, 75]. Therefore, targeting factors associated not only with the acquisition but also with the persistence of oral HPV infection will contribute to both prevention and treatment.

HPV is a small DNA virus with a specific tropism for squamous epithelia. More than 120 different HPV types have been isolated to date. Low-risk HPVs, including HPV-6 and HPV-11, induce benign hyperproliferations of the epithelium such as papillomas or warts which rarely progress to cancer [76]. High-risk oncogenic types such as HPV-16 and HPV-18 are associated with squamous cell carcinoma. HPV-16 and HPV-18 are capable of transforming epithelial cells. This transforming potential is largely a result of the function of two viral oncoproteins, E6 and E7, which inactivate two tumor suppressor proteins, p53 and pRb, respectively. Expression of E6 and E7 results in cellular proliferation, loss of cell cycle regulation, impaired cellular differentiation, increased frequency of mutations, and chromosomal instability [76, 77].

Population-based studies estimate that the prevalence of HPV DNA in normal oral mucosa is 5–10% [14, 76]. In contrast with cervical cancer, in which HPV is present 100% of the time, HPV is present at a smaller and variable percentage in HNSCC, depending on the subsite and the geographic region studied. In a review of 60 studies, the prevalence of HPV DNA was 23.5% in oral cavity SCC, 35.6% in oropharyngeal SCC, and 24.0% in laryngeal SCC [78]. HPV-16 accounted for a larger majority of HPV-positive oropharyngeal SCC (86.7%) compared with HPV-positive oral cavity SCC (68.2%) and HPV-positive laryngeal SCC (69.2%). Conversely, HPV-18 was rare in HPV-positive oropharyngeal SCC (2.8%) compared with HPV-positive oral cavity SCC (34.1%) and HPV-positive laryngeal SCC (17.0%). Aside from HPV-16 and HPV-18, other oncogenic HPV DNAs were rarely detected in HNSCC [78]. In a multi-national study conducted by the International Agency for Research on Cancer (IARC), only 18% of oropharyngeal tumors were HPV-positive [4]. In the majority of recent studies, >50% of oropharyngeal SCC contained the HPV DNA [10–14]. Probability of HPV DNA being detected in the oral mucosa increases with increasing degree of dysplasia. Overall, HPV DNA is 2- to 3-times more likely to be detected in premalignant lesions and about 5-times more likely to be detected in HNSCC compared to normal oral mucosa [79].

HPV-positive HNSCCs have a unique risk factor profile. These tumors are more common in younger patients, have a male predominance, are often staged higher, yet have a survival advantage. These patients are less likely to have used tobacco and alcohol excessively [14]. Pathologically, they are more likely to be basaloid type (poorly differentiated) and lack keratinization. The p16 protein, also known as cyclin-dependent kinase 4 (CDK4) inhibitor, is upregulated in HPV-positive HNSCC, and may serve as a surrogate marker for HPV transcriptional activity. Molecularly, these tumors expresses oncoproteins E6 and E7 demonstrate less chromosomal disruption than HPV-negative HNSCC. Tumor suppressor genes p53 and pRb are usually intact, but the expressed proteins are inactivated by the viral oncoproteins E6 and E7, respectively. In contrast, HPV-negative HNSCC, which is usually initiated by smoking and alcohol, shows considerable chromosomal aberration, including mutation of the p53 gene in 50% of cases [80–82].

Although the natural history of cervical HPV infection is well established, the mechanism of transmission for oral HPV infection, which has a distinct immunologic environment, is largely unknown. There is increasing evidence that oral HPV transmission may be related to sexual history, and particularly oral sexual behavior. Case-control studies have shown associations of oral HPV infection with the number of oral and genital sexual partners, age at first of oral-genital contact, frequency of sexual contacts, and history of genital warts [83–85]. Vertical transmission of oral HPV infection through maternal milk is also possible [86]. HPV infection at each anatomic site is localized and concordance of oral and genital HPV infection seems to be low [14] implying the importance of local oral factors. Research evaluating the role of local oral factors on natural history of oral HPV infection is lacking and is critical to develop effective prevention strategies.

Few epidemiological studies suggest that chronic inflammation of the cervix increases the risks of HPV infection and cervical cancer [87–90]. In addition, molecular studies have shown that inflammatory cytokines, including IL-1, IL-6, and TNF-*α*, modulate proliferation of HPV and expression of its oncogenes E6 and E7 in cervical epithelial cells [91–96]. Supporting these findings, an *in vitro *study has shown that a nonsteroidal anti-inflammatory drug strongly induced degradation of the HPV oncoproteins causing growth arrest and apoptosis in cervical carcinoma cells in a concentration-dependent manner [97]. Continuous expression of the HPV oncoproteins has also been shown to be critical for the maintenance of malignant phenotype in HPV-positive oropharyngeal cancers [98]. The oral cavity and cervix are lined with similar types of mucosa, and the same HPV types cause cervical and head and neck cancers. In a recent hospital-based case-control study with 30 incident primary base of tongue SCC, a history of chronic periodontitis was associated with tumor HPV status after adjustment for age at diagnosis, gender, race/ethnicity, alcohol use, smoking status, ABL, and number of missing teeth (OR = 3.96, 95% CI = 1.18–13.36) (Table 4) [99].

An association between chronic inflammation and HPV infection is biologically plausible. HPV infects basal cells of the epithelium exclusively, and it usually gains access through microabrasions. In addition, replication of the virus is closely associated with basal cell proliferation [74, 75]. Mucosal injury and consequent breaks within the oral mucosa mediated by inflammatory cytokines may facilitate the acquisition as well as the persistence of oral HPV infection [70]. In the presence of chronic inflammation, basal cell proliferation is also increased leading to higher viral load in saliva as well as higher risk of transmission [75]. The observed association between periodontitis and oral HPV infection may be alternatively explained by the direct effects of bacteria [100]. Prospective cohort studies have shown that concurrent infection with *Chlamydia trachomatis* increased the persistence of cervical HPV infection and the risk of cervical cancer [101, 102]. Periodontal bacteria, derived from periodontal pockets, are extensively present in the oral mucosa [103, 104]. However, whether the association between periodontitis and oral HPV infection is through direct effects of bacteria or through stimulation of inflammation is yet to be determined. It is likely that both mechanisms are involved but the bulk of the current evidence suggests that the periodontal bacteria travel from affected tissues to distant sites via saliva and bloodstream and cause tissue injury through inflammatory reactions [105–109].

## 4. Implications for Prevention and Treatment

**Figure 1:**
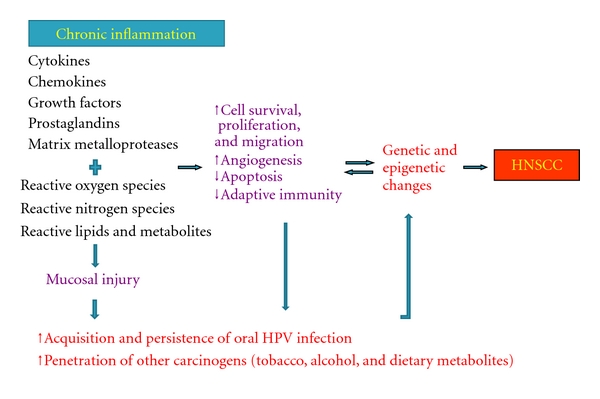
Model for the role of chronic inflammation in the etiology of head and neck squamous cell carcinoma (HNSCC).

The potential role of chronic inflammation in the etiology of HNSCC is summarized in Figure 1. Briefly, chronic inflammation may increase the risk of HNSCC both independently and by facilitating the acquisition and persistence of oral HPV infection.

Understanding the interactions among risk factors may be the most important step towards effective prevention and treatment of HNSCC. It is well established that carcinogenesis is a multifactorial process and the presence of a single risk factor is usually not sufficient to cause cancer. Yet, most studies have focused on independent effects of specific risk factors [6, 7]. Recently, two separate entities, “tobacco-related” and “HPV-related” HNSCC, have been suggested [80–82]. However, this classification overlooks the fact that most HPV-positive HNSCC patients also have a history of tobacco use. It is more likely that the interaction between different combinations of risk factors in each individual determines the disease outcome or its clinical presentation. For example, in our study population consisting of newly diagnosed base of tongue cancer patients, 83.5% had a smoking history and 63.3% had HPV-positive tumors. The majority (71.4%) of patients with HPV-positive tumors also had a smoking history [99]. The results further suggested that smokers with chronic periodontitis were more likely to have HPV-positive tumors, and smokers without periodontitis history were more likely to have HPV-negative tumors [99]. Smoking is a strong risk factor for chronic periodontitis [21]. Although validation with further studies is needed, these observations suggest that only a subset of smokers who develop chronic inflammatory diseases (perhaps long-term, moderate to heavy smokers) are at increased risk for oral HPV infection.

Saliva provides a means of interaction between different carcinogens. It also provides a means of transport from one surface to another promoting disease at distant sites. Salivary enzymes also metabolize tobacco, alcohol, and dietary components into carcinogens [110]. This may contribute to the field cancerization in the head and neck region. The fact that the majority of HNSCC occurs at saliva-draining areas supports this hypothesis [111]. While periodontitis is localized to the structures around teeth, periodontal pocket contents, including bacteria, viruses, inflammatory cytokines, enzymes, and toxins are continuously shed into saliva [22, 23, 112]. On the other hand, saliva has no access into periodontal pockets. Therefore, periodontal pockets are probably a source of inflammatory and microbial markers in saliva rather than being the actual sites of carcinogenesis.

It is critical to understand the characteristics of the dental biofilm to effectively treat periodontitis. Biofilms are sessile communities of bacteria embedded in a polymeric matrix irreversibly attached to a surface. The bacteria in the biofilm differ profoundly from their free-floating counterparts and are resistant to antimicrobials as well to host defense [113]. Thus, effective treatment of periodontitis is based on mechanical removal of the biofilm. In the initial stages of the disease, a thorough professional dental cleaning and maintenance of a good personal oral hygiene, by brushing and flossing, is usually sufficient to reverse the disease process. In more advanced stages, however, the biofilm or calculus in periodontal pockets is inaccessible by personal oral hygiene practices. In these cases, surgical reduction of the periodontal pockets and/or periodic professional cleanings may be necessary to control the disease [19, 113].

If prospective studies confirm that chronic local inflammation is in fact a significant player in the natural histories of oral HPV infection and HNSCC, this may have important implications, not only for prevention, but also for treatment. Chronic local inflammation, such as periodontitis, is easy to detect and may represent a clinical high-risk profile for both oral HPV infection and HNSCC. Subjects with sources of chronic inflammation in the oral cavity may be screened for oral HPV infection and HNSCC for early diagnosis.

## 5. Conclusion

Chronic local inflammation, both independently and by facilitating oral HPV infection, may be an important player in the etiology of HNSCC.

## Figures and Tables

**Table 1:** Chronic inflammatory diseases and associated cancers.

Chronic inflammation	Associated neoplasm
Chronic bronchitis	Lung carcinoma
Chronic gastritis	Gastric adenocarcinoma
Chronic cervicitis, pelvic inflammatory disease	Cervical carcinoma, ovarian carcinoma
Asbestosis, silicosis	Mesothelioma, lung carcinoma
Chronic ulcerative colitis	Colorectal cancer
Chronic pancreatitis, hereditary pancreatitis	Pancreatic carcinoma
Chronic sunburned skin, skin inflammation	Melanoma, basal-cell carcinoma, squamous cell carcinoma
Reflux esophagitis, Barrett's esophagus	Esophageal carcinoma
Chronic hepatitis	Hepatocellular carcinoma
Mononucleosis	Burkitt's, Hodgkin's and non-Hodgkin's lymphoma.
Chronic cystitis	Bladder cancer
Chronic osteomyelitis	Skin carcinoma in draining sinuses

Table compiled from [15–18].

**Table 2:** Risk of oral premalignant lesions from periodontitis.

	Premalignant lesions*	Any lesion
*N* (%)^†^	323 (2.3%)	3,421 (24.8%)
Periodontitis^‡^	1.55 [1.06–2.27]^§^	1.09 [0.91–1.31]

*Premalignant lesions were defined as erythroplakia, leukoplakia (homogeneous and nonhomogeneous), nonspecific ulcer, and smokeless tobacco-associated lesions.

^†^Count (percentage).

^‡^Periodontitis was defined as CAL >1.5 mm.

^§^Odds ratios (OR) and their 95% confidence intervals derived from multiple logistic regression analysis adjusting for age, gender, race/ethnicity, education, tobacco, alcohol, and occupational hazard.

**Table 3:** Association between periodontitis and head and neck cancer stratified by tumor site (*N* = 473).

Alveolar bone loss (per millimeter)	*N* (cases/controls)	Crude OR [95% CI]*	Adjusted^†^ OR [95% CI]
All HNSCC patients	266/207	3.85 [2.96–5.01]	4.36 [3.16–6.01]
Oral cavity SCC patients	100/207	3.26 [2.44–4.36]	4.52 [3.03–6.75]
Oropharyngeal SCC patients	115/207	3.06 [2.29–4.07]	3.64 [2.54–5.22]
Laryngeal SCC patients	51/207	3.75 [2.60–5.41]	2.72 [1.78–4.16]

*Odds ratios and their 95% confidence intervals.

^†^Adjusted odds ratios were derived from multiple logistic regression analysis including age at diagnosis, gender, race/ethnicity, marital status, smoking status, alcohol use, ABL, and missing teeth.

**Table 4:** Odds Ratios for Tumor HPV Status Associated with Periodontal Variables.

	Crude OR	Adjusted^†^ OR
	[95% CI]*	[95% CI]
Alveolar Bone Loss (per millimeter)	2.86 [1.03–7.98]	3.96 [1.18– 13.36]
Missing Teeth (per tooth)	0.94 [0.86–1.02]	0.95 [0.74–1.21]

*Odds ratios and 95% confidence intervals.

^†^Adjusted odds ratios were derived from multiple logistic regression model including age at diagnosis, gender, race/ethnicity, alcohol use, smoking status, ABL and number of missing teeth.
